# Social pairing of Seychelles warblers under reduced constraints: MHC, neutral heterozygosity, and age

**DOI:** 10.1093/beheco/arv150

**Published:** 2015-09-28

**Authors:** David J. Wright, Lyanne Brouwer, Maria-Elena Mannarelli, Terry Burke, Jan Komdeur, David S. Richardson

**Affiliations:** ^a^School of Biological Sciences, University of East Anglia, Norwich Research Park, Norwich, Norfolk NR4 7TJ, UK,; ^b^Department of Animal and Plant Sciences, NERC Biomolecular Analysis Facility, University of Sheffield, Sheffield S10 2TN, UK,; ^c^Evolution, Ecology & Genetics, Research School of Biology, The Australian National University, Canberra, Australian Capital Territory 0200, Australia,; ^d^Behavioural Ecology and Self-organization Group, Centre for Ecological and Evolutionary Studies, University of Groningen, PO Box 11103, 9700 CC Groningen, The Netherlands, and; ^e^Nature Seychelles, Centre for Environment and Education, The Sanctuary, PO Box 1310, Roche Caiman, Victoria, Mahé, Republic of Seychelles

**Keywords:** compatibility, extrapair paternity, good genes, MHC class I, sexual selection, translocation.

## Abstract

The prevalence and significance of precopulatory mate choice remains keenly debated. The major histocompatibility complex (MHC) plays a key role in vertebrate adaptive immunity, and variation at the MHC influences individual survival. Although MHC-dependent mate choice has been documented in certain species, many other studies find no such pattern. This may be, at least in part, because in natural systems constraints may reduce the choices available to individuals and prevent full expression of underlying preferences. We used translocations to previously unoccupied islands to experimentally reduce constraints on female social mate choice in the Seychelles warbler (*Acrocephalus sechellensis*), a species in which patterns of MHC-dependent extrapair paternity (EPP), but not social mate choice, have been observed. We find no evidence of MHC-dependent social mate choice in the new populations. Instead, we find that older males and males with more microsatellite heterozygosity are more likely to have successfully paired. Our data cannot resolve whether these patterns in pairing were due to male–male competition or female choice. However, our research does suggest that female Seychelles warblers do not choose social mates using MHC class I to increase fitness. It may also indicate that the MHC-dependent EPP observed in the source population is probably due to mechanisms other than female precopulatory mate choice based on MHC cues.

## Lay Summary

We manipulated factors that normally constrain the social mate choice of female Seychelles warblers to test if, when less constrained, these females chose partners based on variation at specific immune genes that have been linked to mate choice in other species. We found that older and more generally genetically diverse males were more likely to end up paired with females, but contrary to our predictions, variation at the immune genes did not appear to influence social pairing.

## INTRODUCTION

The prevalence and significance of precopulatory mate choice remains a keenly debated topic in sexual selection, mainly due to difficulties in quantifying the evolutionary costs and benefits of being “choosy” (Andersson 1994; Kokko et al. 2003). Mate choice can provide both direct benefits, such as superior nest guarding, and indirect benefits, such as increased genetic diversity in offspring (Hamilton and Zuk 1982; Andersson 1994). It can manifest in behavioral patterns, such as the choosing of a social mate, and eventually in genetic patterns, such as bias in offspring genotypes (Jennions and Petrie 2000; Consuegra and Garcia de Leaniz 2008). Genes of the major histocompatibility complex (MHC), which determine antigen recognition in the adaptive immune response of vertebrates (Klein 1986), have been a focus of mate choice research for decades (Yamazaki et al. 1976; Jordan and Bruford 1998; Milinski 2006; Kamiya et al. 2014). Different MHC genotypes confer differential pathogen resistance and, therefore, fitness to individuals (Briles et al. 1977; Ditchkoff et al. 2001; Wedekind et al. 2004). This makes the MHC an obvious candidate for genes that underpin the benefits of mate choice, and several hypotheses have been proposed to explain how individuals may optimize the MHC genotypes that their offspring inherits (Penn and Potts 1999; Milinski 2006; Kamiya et al. 2014).

Various mechanisms have been invoked to explain apparent mating preferences. Individuals may choose mates based on “good genes”: either particular beneficial alleles (a classical “good genes” scenario) or on heterozygosity at specific loci (referred to as “good-genes-as-heterozygosity”), or a combination of the two. When calculated over many duplicated loci this heterozygosity can translate into choice for overall diversity, thus the latter scenario can be referred to as a “diversity” mechanism (Kamiya et al. 2014). In an MHC-dependent scenario, choice under a “good genes” or a “diversity” mechanism may be achieved by assessing indicators of condition such as secondary sexual traits that are linked to MHC characteristics (Hamilton and Zuk 1982; Ditchkoff et al. 2001; Milinski 2006). By choosing a mate with a superior MHC genotype, individuals may obtain direct benefits such as better provisioning for their offspring (Andersson 1994) as a result of the better condition (immunocompetence) of the mate or indirect benefits by providing offspring with specific advantageous alleles and/or increased MHC diversity (Hamilton and Zuk 1982; Reusch et al. 2001). Individuals may also choose mates based on their MHC similarity, in order to obtain an optimal level of MHC diversity in their offspring (Nowak et al. 1992). Mate choice under such a “compatibility” mechanism is based on indirect benefits. What constitutes a “good match” depends on the complementarity of the maternal and paternal genotypes (Yamazaki et al. 1976; Milinski 2006), although maximizing dissimilarity with a mate may not necessarily be the best strategy if there could be negative consequences of too high a level of MHC diversity (e.g., Milinski 2006; Kalbe et al. 2009). Importantly, as well as the ability to assess the MHC characteristics of others (e.g., via olfaction), “compatibility” mechanisms require self-recognition, so that individuals can gauge their compatibility with potential mates (Penn 2002; Milinski 2006). These mate choice models are normally viewed from the female perspective, though male choice is important in some systems (see Gillingham et al. 2009; Edward and Chapman 2011). Finally, MHC genes may act as markers of relatedness and be used to avoid close inbreeding, rather than to acquire specific MHC characteristics per se (Brown and Eklund 1994; Penn and Potts 1998).

Numerous studies have investigated MHC-dependent pairing and fertilization patterns providing evidence for each of the different hypothesized mechanisms in different species (e.g., Penn and Potts 1999; Kokko et al. 2003; Andersson and Simmons 2006; Milinski 2006; Kotiaho and Puurtinen 2007; Griggio et al. 2011; Løvlie et al. 2013; Kamiya et al. 2014). However, many other studies find no evidence of MHC dependence (e.g., Paterson and Pemberton 1997; Westerdahl 2004; Huchard et al. 2010), and the prevalence of MHC-dependent mate choice is unclear. Although many taxa may simply not have evolved mechanisms of MHC-dependent mate choice, its absence in a population may also be due to constraints on choice. Constraints may occur to some extent in almost all species (Arnqvist and Rowe 2005) due to factors such as social monogamy (Cohas et al. 2006) or forced pairings (Casalini et al. 2009). Significant research has focused on the consequences of constraints for the evolution of alternative mating strategies such as reproductive compensation and promiscuity (Cohas et al. 2006; Gowaty et al. 2007; Setchell and Huchard 2010), but the implications of mate choice constraints for MHC diversity in wild populations remain unclear.

Here, we take an experimental approach to investigate whether a reduction of constraints leads to the expression of MHC-dependent social mate choice in the Seychelles warbler (*Acrocephalus sechellensis*). This socially monogamous species was previously restricted to a single island, Cousin, where the population has been at carrying capacity since 1982 (Brouwer et al. 2009). On Cousin, a combination of habitat saturation, longevity, and social fidelity is thought to severely constrain social mate choice (Richardson et al. 2005), indeed around 29% of adults never manage to obtain a breeding territory/position (Komdeur 1991). However, Seychelles warblers are highly promiscuous, with extrapair paternity (EPP) accounting for circa 40% of offspring (Richardson et al. 2001). This promiscuity is linked to MHC class I variation: females are more likely to have EPP if their social male is of low MHC diversity and do so with extrapair males that are more MHC diverse (Richardson et al. 2005). Consequently, this EPP improves the MHC diversity and thus survival of the female’s offspring (Brouwer et al. 2010). Previous findings do not allow us to discern the mechanism generating these MHC-dependent fertilization patterns as they are consistent with both male–male competition—with less diverse MHC males less able to mate guard or acquire EPP—and active female choice for MHC diverse EPP males. We created an opportunity to test for MHC class I dependent social pairings under natural conditions by using translocations of Seychelles warblers to 2 new islands as part of the long-term conservation of this species (Richardson et al. 2006; Wright et al. 2014). Significantly more males than females were translocated to each island, where a large surfeit of optimal habitat meant that each male was able to establish a high-quality territory compared with those inhabited on the original island. Consequently, females had the opportunity to pair up with any one of multiple males, all with high-quality territories. Thus, we provide a relatively unconstrained arena in which to test whether specific social mating patterns occur and to assess whether such patterns may be driven by active female mate choice or other mechanisms. Given the offspring survival benefits resulting from mating with a MHC diverse male, we predict MHC class I characteristics will play an important role in any social mate choice in these populations. Under a “diversity” mechanism (i.e., good-genes-as-heterozygosity), we expect females to prefer males with high MHC diversity. Under a classical “good genes” mechanism, we would expect a link between male pair status and individual MHC class I alleles, if social mate choice is based on the presence of specific alleles—such as *Ase-ua4*, which has previously been shown to influence survival (Brouwer et al. 2010). Under a “compatibility” mechanism females would pair with maximally or optimally (Milinski 2006) MHC dissimilar males. Finally, we test whether stability of these pair bonds is MHC dependent. If “diversity” is important, males of low MHC diversity are more likely to suffer the subsequent breaking of any established pair bonds (divorce) than higher diversity males. If “compatibility” is important, pairs that divorce are expected to be more MHC similar than pairs that remain stable.

## MATERIALS AND METHODS

### Study populations

Each translocation was performed as outlined by Richardson et al. (2006), with all birds caught on Cousin (4°21′S 55°38′E, 0.29 km^2^). Translocation of existing pairs was avoided where possible, although a small number were transferred (Denis = 7, Frégate = 1, see Results for details). However, previous work (Komdeur 1996) showed that birds paired in the source population did not normally re-pair in the new populations in previous translocations. Individuals were identified with a unique combination of colored leg rings. A 25-µL blood sample was taken from each bird by brachial venipuncture and stored in absolute ethanol. A total of 58 birds (34 males, 24 females) were moved to Denis (3°48′S 55°40′E, 1.42 km^2^) in 2004 (Richardson et al. 2006) and 59 birds (36 males, 23 females) to Frégate (4°35′S 55°56′E, 2.19 km^2^) in 2011 (Wright et al. 2014). Each translocation was undertaken in 2 batches on different days (Denis: *n* = 35 on 30 May 2004, *n* = 23 on 12 June 2004; Frégate: *n* = 22 on 07 December 2011, *n* = 37 on 14 December 2011). All individuals were released at the same site on each island. Age at translocation (determined in reference to hatching year estimated at first capture) was classified into “young” (<2 years) and “old” (>2 years), following Brouwer et al. (2006). There was no bias in age of individuals in each batch and catching was undertaken across the entire island by multiple catching teams, covering all areas simultaneously (Richardson et al. 2006; Wright et al. 2014). Each new population was censused and monitored for breeding up to 3 months postrelease (Denis: continually from release until August 2004, Frégate: February 2012) and again after 1 year postrelease (Denis: May–August 2005, Frégate: March–May 2013).

### Social mate choice

Territories were mapped on each new island. The average territory size of Seychelles warblers on Cousin is 230 m^2^ (Brouwer et al. 2009). With at least 300000 m^2^ of suitable habitat on Denis (Richardson et al. 2006) and 390000 m^2^ on Frégate (Richardson and Hammers 2011), the spatial and habitat quality constraints that existed in the original population were greatly reduced (if not totally removed) for males released on the new islands. Individuals were located and followed for a minimum of 15min repeatedly (ca. weekly) during the field period, during which interactions with other conspecifics were recorded. After a pair bond and territory are established, Seychelles warblers display a specific repertoire of behaviors that allows clear classification of pair status (Richardson et al. 2003). Previous studies found that although a few pairs may form and nest within days of translocation, most stable pairings only formed after 2 months of rapid switching (Komdeur 1996). Hence, social mate choice was assessed after 3 months and by multiple observations where possible. Pairings were reassessed 1 year after the initial social pairing observations. Divorce was identified when both individuals were resighted, with at least 1 individual in a new pair. In cases where one of a pair was not resighted over consecutive fieldwork periods, it was assumed that individual had died and the pairing was considered faithful until death. Any subsequent pairing of the surviving individual was not considered the result of divorce and was excluded from analysis, but we acknowledge this is a conservative measure.

### Molecular analyses

Samples were genotyped at 30 microsatellite loci following Spurgin et al. (2014). We tested for deviations from Hardy–Weinberg equilibrium and for linkage disequilibrium between loci using GENEPOP v. 4.1 (Raymond and Rousset 1995). Null allele frequencies were estimated using CERVUS v. 3.0 (Marshall et al. 1998). Three estimators of pairwise relatedness *r* (Queller and Goodnight 1989; Lynch and Ritland 1999; Wang 2002) were calculated in COANCESTRY v. 1.0 (Wang 2011). These 3 estimators were highly correlated (Mantel tests, all *r* ≥ 0.80), and the results remained consistent regardless of the estimate used, therefore only Queller and Goodnight’s *r* is reported. Standardized individual microsatellite heterozygosity (Hs) was calculated using the R package Genhet ( Coulon 2010 ).

Variation at exon 3 of MHC class I (which codes for the peptide-binding region [PBR] involved in antigen recognition) was screened using reference-strand–mediated conformation analysis (RSCA) and the primer sets from Richardson and Westerdahl (2003), following the method of Worley et al. (2008). Each segregating RSCA variant corresponded to a unique 255 base pair amino acid coding sequence (of a total exon length of 274 base pairs, Richardson and Westerdahl 2003). A total of 10 MHC class I variants have been detected in the Seychelles warbler, with individuals possessing between 2 and 8 variants each, suggesting that at least 4 class I loci are amplified (Richardson and Westerdahl 2003). Although it is impossible to identify which locus each variant comes from, they are hereafter termed “alleles” for simplicity. This method does not provide a measure of locus-specific heterozygosity, but an overall estimate of MHC class I exon 3 diversity, which will correlate with heterozygosity across the amplified loci. This heterozygosity measure has shown to be an important parameter linked to fertilization patterns and survival in the Seychelles warbler (Richardson et al. 2005; Brouwer et al. 2010). Previous work by Hutchings (2009) detected no variation at MHC class II loci in the Seychelles warbler and so these loci were not assessed in the current study.

MHC-dependent mate choice may be based on only the functional differences between alleles. To address this, codons comprising the PBR were superimposed onto the Seychelles warbler sequences (see Richardson and Westerdahl 2003). However, these PBR codons were identified in humans (Bjorkman et al. 1987) and while strongly conserved across taxa, may not be completely accurate in the Seychelles warbler. Therefore, another way to determine functional differences between alleles is to compare between sites that have been identified as being under positive selection (positively selected sites [PSS]) in passerines. To identify these PSS, MHC class I exon 3 sequences from a range of passerine genera were downloaded from NCBI GenBank: *Acrocephalus* (*n* = 16), *Passer* (*n* = 38), *Parus* (*n* = 64, of which *Cyanistes* = 59), and *Carpodacus* (*n* = 28) and aligned to the Seychelles warbler (*n* = 10) in BIOEDIT v. 7.1 (Hall 1999). Three methods were employed to detect positive selection. Single likelihood ancestor counting (SLAC) and fast unbiased Bayesian approximation (FUBAR) are maximum likelihood methods that estimate the nonsynonymous to synonymous substitution rate (d*N*/d*S* ratio, ω) at each codon, the latter utilizing an Markov chain Monte Carlo approach to increase model accuracy (Kosakovsky Pond and Frost 2005; Murrell et al. 2013). The third method—the mixed effects model of evolution (MEME)—identifies episodic bouts of positive selection across an alignment by allowing ω to vary by codon and branch within the phylogeny (Murrell et al. 2012). Each method was implemented under the conservative general time reversible model and neighbor-joining tree with probabilities of <0.05 (SLAC and MEME) and posterior probability of ≥0.95 (FUBAR) using HYPHY (Kosakovsky Pond et al. 2005) and a web-based user interface operating on a remote cluster available at http://www.datamonkey.org (Delport et al. 2010). Only codons identified by all 3 methods were accepted as putatively PSS.

### Statistical analyses

Statistical analyses were performed in R v. 2.15 (R Development Core Team 2012) unless otherwise stated. Throughout, the term “pairs” denotes observed pairings, and “dyads” all other male–female pair combinations possible in each analysis. All comparisons of pairs and dyads were performed using randomization tests (Manly 1997) in MSEXCEL plug-in POPTOOLS v. 3.2 (Hood 2010). This is a useful approach to testing whether observed data differ significantly from random expectation. In each instance detailed below, the difference between observed pairs and all other possible male–female dyad combinations was tested with analysis of variance (Anova). The data were then resampled without replacement (shuffled) and retested 10^5^ times. Estimates of significance were calculated as the proportion of repetitions in which the resampled Anova *F* value was equal to, or exceeded, the test Anova *F* value. This approach was employed to control for any effect of inbreeding avoidance on social mate choice, by comparing mean relatedness (*r*) of pairs and dyads for each island. To investigate “good-genes-as-heterozygosity” diversity predictions, the probability of a male being paired was analyzed using a generalized linear model with a binomial error structure and logit-link function, with number of MHC alleles and standardized individual heterozygosity (Hs) as covariates, and island and age class as categorical fixed factors. Data from both islands were combined to maximize sample size, hence inclusion of “island” as a fixed factor in the model. Model fit was assessed by comparison against the null model (constant only) and Nagelkerke’s *R* ^2^. We also tested for an interaction between individual MHC alleles and male pair status under a classical “good genes” scenario using Fisher’s Exact tests, with correction for multiple testing. MHC similarity between pairs and dyads was calculated in 2 ways. First, the proportion of alleles shared (*S*_*xy*_), which is double the number of alleles shared between 2 individuals, divided by the sum of each individual’s alleles [*S*_*xy*_ = 2*N*_*xy*_/(*N*_*x*_ + *N*_*y*_)] (Wetton et al. 1987). Second, amino acid divergence (*p*-distance) was calculated between each MHC allele sequence for codons involved in the PBR and (separately) also for codons identified as putative PSS. Amino acid *p*-distances were calculated in MEGA v. 5.1 (Tamura et al. 2011), and the mean pairwise amino acid divergence between pairs and dyads was calculated. Comparisons of each measure of complementary (*S*_*xy*_, PBR, and PSS) were then conducted using randomization tests as described previously. Mate choice under a compatibility mechanism may be masked if the optimal similarity between pairs lies close to that observed in random dyads. In this case, observed pairs are predicted to show less variation around this mean similarity than random dyads. Therefore, we tested variance in MHC similarity of observed pairs versus simulated dyads using the same randomization approach. The association between the MHC similarity of a pair and the likelihood of divorce was tested using Mann–Whitney–Wilcoxon tests on divorced versus faithful pairings, using each measure of MHC similarity (*S*_*xy*_, PBR, and PSS). Finally, we tested for differences in MHC diversity between divorced and stable-pair individuals of each sex using the randomization approach.

## RESULTS

All individuals were released unharmed in both translocations. Three months postrelease, 56 out of 58 birds (97%) were resighted on Denis and 50 out of 59 birds (85%) on Frégate. Annual survival of adult Seychelles warblers is high (84%; Brouwer et al. 2006), but unpaired birds are more difficult to locate than paired birds due to aggressive territoriality in the latter. All but one male individual was resighted on Denis in subsequent years, and we assumed similar survival on Frégate (indeed, 92% [54/59] of individuals were seen in the subsequent year on Frégate). For this reason, individuals not seen during the initial study period (Denis = 2 males, Frégate = 3 females and 6 males) were treated as alive and unpaired. A total of 40 pairings was confirmed in the 2 translocated populations: 40 males were considered paired and 30 unpaired. MHC data were unavailable for 3 individuals (Denis = 2, Frégate = 1), and these were excluded from analyses along with their pair bird, leaving 37 pairings (Denis = 19, Frégate = 18). Eight existing pairs from Cousin were translocated (Denis = 7, Frégate = 1), but only 2 of these pairs re-paired once released (both on Denis). Observations of pairs 1 year after revealed that 29 pairs had remained together and 8 had divorced.

### Genetic markers

Microsatellite and MHC class I genotypes were compiled for all 111 individuals included in the analyses (Denis = 54, Frégate = 57). Neither population showed significant departure from Hardy–Weinberg equilibrium at any microsatellite locus. Linkage disequilibrium was detected between 1 loci pair in Denis (*Ase*-13 and *Ase*-48, *P*_crit_ = 0.0001) and between 2 loci pairs in Frégate (*Ase*-56 and *Ase*-38, *Ase*-48 and Cuu4-gga, both *P*_crit_ = 0.0001) after Bonferroni correction. Null allele frequencies of 0.11 were detected at loci *Ase*-56 and *Ase*-38 in the Frégate population, but −0.013 and −0.096, respectively, in the Denis population. Analyses repeated with and without one of each pair of loci showed no qualitative difference (data not shown) and so all loci were retained.

There was no difference in the number of MHC class I alleles in each sex (females: *n* = 44, mean ± standard error [SE] = 4.70±0.22 vs. males: *n* = 67, mean ± SE = 4.61±0.17, *W* = 1462.5, *P* = 0.94) or between islands (Denis = 4.77±0.18 vs. Frégate = 4.52±0.20, *W* = 1362, *P* = 0.28), and all 10 alleles were present in each population. Selection tests indicated that 7 of the 85 codons of exon 3 were putatively PSS, of which 3 corresponded to the 7th, 9th, and 11th codons of the superimposed PBR (Supplementary Figure S1).

### Social mate choice

#### Inbreeding avoidance

Pairwise relatedness (*r*) of social pairs varied between *r* = −0.31 and 0.41 (mean ± SE = 0.02±0.03) across both populations with 5 pairs being related to the degree of half-sib or higher (*r* > 0.25). No difference was detected between the relatedness of pairs versus random dyads on either island (randomization tests: Denis 19 pairs vs. 342 dyads, *P* = 0.40 and Frégate 18 pairs vs. 306 dyads, *P* = 0.16).

#### Good-genes-as-heterozygosity

**Figure 1:**
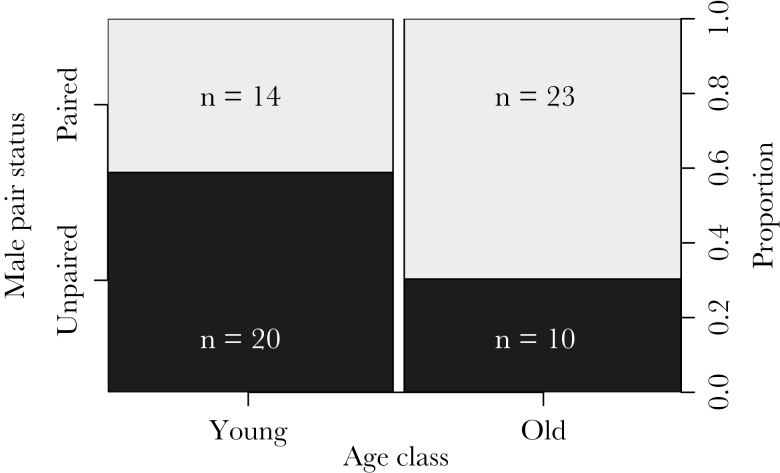
Proportions of male Seychelles warblers paired (light gray) and unpaired (dark gray) in 2 newly established populations for each age class, 3-month postrelease: young (<2 years, *n* = 34) and old (>2 years, *n* = 33). Data are combined from both translocated populations.

**Figure 2:**
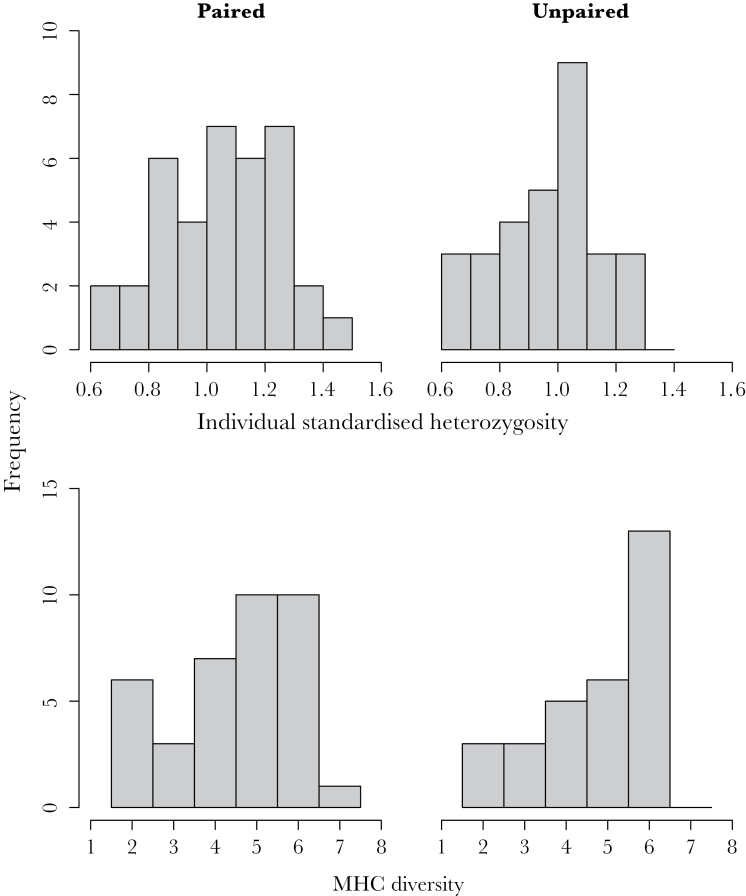
Frequency histograms of individual standardized heterozygosity (upper) and MHC class I diversity (lower) in paired (*n* = 37) and unpaired (*n* = 30) male Seychelles warblers in the newly established populations 3-month postrelease.

The full generalized linear model (Table 1) was a significant improvement on the null model (χ^2^ = 10.59, degrees of freedom = 4, *P* = 0.03). No collinearity was detected between predictors (all tolerances ≥ 0.88). MHC diversity did not predict whether males were paired or not (Table 1), with a mean (± SE) MHC diversity of paired males = 4.49±0.24 and unpaired males = 4.77±0.25. Age class and, to a lesser degree, individual neutral heterozygosity, were both significant predictors of male pair status (Table 1). Older males (*n* = 33) were more likely to be paired than younger males (*n* = 34, 69.7% vs. 41.2%, respectively, Figure 1) and paired males (*n* = 37) were generally more heterozygous than unpaired males (*n* = 30, mean ± SE of 1.04±0.03 vs. 0.95±0.03, respectively, Welch’s *t*_64.83_ = 1.94, *P* = 0.05, Figure 2), but this was not due to older males being more heterozygous (older males mean ± SE = 1.00±0.03 vs. young males = 1.00±0.03; Welch’s *t*_64.83_ = 0.02, *P* = 0.98). Similarly, MHC diversity did not differ between male age categories (older males mean ± SE = 4.54±0.24 vs. young males = 4.68±0.25, *W* = 563, *P* = 0.98). Including which transfer batch (date) individuals were moved, or testing for a quadratic effect of male MHC diversity did not significantly increase the explanatory power of the model and did not influence the effects of age and microsatellite heterozygosity on male pairing.

**Table 1:** Generalized linear model with a binomial error structure and logit-link function, predicting the pairing status of male Seychelles warbler within the newly established populations in relation to MHC class I diversity, age class (young or old), individual standardized heterozygosity (Hs), and island (Denis or Frégate)

Predictors	*B* (SE)	χ^2^	df	95% CI for odds ratio			*P* value
				Lower	Odds ratio	Upper	
Constant	−2.49 (1.72)	−1.45	1				
MHC diversity	−0.19 (0.19)	−0.98	1	0.56	0.83	1.2	0.32
**Age class**	**1.27 (0.57**)	**2.23**	**1**	**1.2**	**3.55**	**11.38**	**0.02**
**Hs**	**2.95 (1.46**)	**2.02**	**1**	**1.23**	**19.16**	**413.1**	**0.04**
Island	0.03 (0.56)	0.05	1	0.34	1.03	3.22	0.96

Maximum model χ^2^ = 10.59, df = 4, *P* = 0.03, and *R* ^2^ = 0.20 (Nagelkerke). Parameters significant at *P* < 0.05 are highlighted in bold. CI, confidence interval; df = degrees of freedom.

#### Classical good genes

Fisher’s Exact tests showed 1 allele, *Ase-ua3*, had a weak negative relationship with male pair status (i.e., the presence of *Ase-ua3* meant lower probability of being paired) before correction for multiple testing (*P* = 0.03, odds ratio: 0.26), but there were no significant interactions between any of the individual MHC class I alleles and male pair status after Bonferroni correction for multiple testing (*P*_crit_ = 0.005).

#### MHC compatibility

There was no difference in mean MHC allele sharing (*S*_*xy*_) between pairs (*n* = 19) and random dyads (*n* = 342) on Denis (mean ± standard deviation: pairs = 0.64±0.30 vs. dyads = 0.64±0.25, randomization test *P* = 0.95) or between pairs (*n* = 18) and random dyads (*n* = 306) on Frégate (pairs = 0.58±0.28 vs. dyads = 0.50±0.28, *P* = 0.22). Furthermore, there was no difference in the variance between pairs and random dyads (Denis *P* = 0.87, Frégate *P* = 0.28, details above). Similarly, there was no difference in mean amino acid divergence (*p*-distance) between pairs versus random dyads for either the PBR codons (Denis pairs = 0.36±0.026 vs. dyads = 0.36±0.031, randomization tests *P* = 0.69 and Frégate pairs = 0.33±0.05 vs. dyads = 0.35±0.04, *P* = 0.19) or the PSS codons (Denis pairs = 0.38±0.037 vs. dyads = 0.38±0.039, *P* = 0.89 and Frégate pairs = 0.34±0.07 vs. dyads = 0.36±0.06, *P* = 0.14).

#### Divorce

Males that divorced (*n* = 8) within 1 year after translocation had lower MHC diversity than males of stable (*n* = 29) pairs (mean ± SE: divorced = 3.37±0.46 vs. stable = 4.79±0.25, randomization test *P* = 0.02). However, there was no difference in MHC diversity of stable or divorced females (divorced = 4.13±0.74 vs. stable = 4.79±0.25, randomization test *P* = 0.30). Similarly, no difference was detected between divorced versus stable pairs with any of the measures of MHC similarity: *S*_*xy*_ (divorced = 0.59±0.10 vs. stable = 0.61±0.05, *W* = 128, *P* = 0.67), PBR *p*-distance (divorced = 0.32±0.02 vs. stable = 0.35±0.01, *W* = 165.5, *P* = 0.10), or PSS *p*-distance (divorced = 0.30±0.03 vs. stable = 0.37±0.01, *W* = 161.5, *P* = 0.07).

## DISCUSSION

We found no evidence that the occurrence of social pairings in founding populations of Seychelles warblers was influenced by MHC class I characteristics, that is, male MHC diversity, specific alleles, or male–female MHC compatibility within the new island populations. However, older and more neutrally heterozygous males were more likely to be paired. Even when constraints on female choice were greatly reduced, the MHC-dependent patterns of extrapair fertilizations observed in the original source population did not translate into MHC-dependent social mate choice in the new populations. Furthermore, we show that the relatedness of 5 pairs actually exceeded half-sibship (*r* > 0.25) suggesting little attempt or ability even to avoid inbreeding, a result consistent with previous work on the source population (Richardson et al. 2004; Eikenaar et al. 2008).

Divorced males appeared to be less MHC diverse than those in stable pairings. This pattern is consistent with the previous work on MHC-dependent EPP (Richardson et al. 2005) in which less MHC diverse males were cuckolded by more MHC diverse males. The small sample size (8 divorced, 29 stable) means it should be interpreted with caution and we do not possess data on the new pairings of divorced birds for comparison. These data do not enable us to distinguish between active female choice to leave, or male–male competition/male condition, where a male might be less able to defend a mate or territory from a rival (e.g., Hasselquist and Sherman 2001). It does, however, point to a possible link between pair stability and male MHC diversity worthy of future investigation. A slight trend was observed between divorce and MHC PSS (*P* = 0.07) that suggests pairs with more similarity at PSS were more likely to divorce than those more different at PSS. Although this is intriguing, we acknowledge that this may just be a spurious tendency and statistical power and further inference is limited.

The lack of an association between MHC class I diversity or specific alleles and social mate choice suggests that MHC-dependent social mate choice does not occur in this species, a result that concurs with that observed in the congeneric great reed warbler (*Acrocephalus arundinaceous*) (Westerdahl 2004) and other passerines such as the great tit (*Parus major*) (Sepil et al. 2015). The importance of recognition mechanisms such as olfaction in avian reproductive behavior is now widely acknowledged (Caro et al. 2015), and MHC-dependent mate choice suggestive of an olfactory capability has been reported in blue petrels (*Halobaena caerulea*) (Strandh et al. 2012). However, to our knowledge, a mechanism allowing self-recognition and assessment of MHC compatibility has not yet been reported in passerines. Alternatively, subtle patterns of MHC-dependent social mate choice may not have been detected owing to a limited sample size (*n* = 37 pairs). However, the complete distribution overlap of MHC diversity of paired and unpaired males within the dataset (Figure 2) suggests the lack of a significant result is not due to a type 2 error, and similar samples sizes have detected MHC-dependent patterns in other studies of wild populations (Bonneaud et al. 2006; Juola and Dearborn 2012). The accuracy of likelihood selection methods (Anisimova et al. 2003) and thus the identification of PSS could have been reduced by recombination because complex recombination (e.g., gene conversion) can occur at the MHC region (Wittzell et al. 1999; Spurgin et al. 2011). Therefore, we acknowledge that the conservative tests employed here could have misidentified or missed PSS thus weakening our ability to detect choice based on these sites. However, because our previous work found associations with MHC class I diversity and fertilization, we suggest that the absence of MHC-dependent social mate choice, even when choice constraints have been much reduced, is the most likely explanation of our findings.

Older and more heterozygous males were more likely to be paired than younger and less heterozygous males, but this was not due to more heterozygous males being older. The finding that heterozygous males being were more likely to be paired could be explained by mate choice under a “good-genes-as-heterozygosity” diversity model, which has been reported in birds (Kempenaers 2007). The presence of closely related pairs in the new populations suggests that male–female complementarity does not play a role in this. Indeed, as Seychelles warbler pair bonds last over multiple breeding attempts, overall male quality (i.e., heterozygosity) may be a more important factor than diversity at specific genes. Age-dependent social mate choice (Kokko and Lindstrom 1996; Kokko 1998) and extrapair fertilization success (Richardson and Burke 1999; Cleasby and Nakagawa 2012) have both been observed in various species. Even though Seychelles warblers can breed successfully at 8 months of age (Komdeur 1992), territory acquisition is age related, with older males more likely to gain a breeding territory than younger ones, probably mediated by male–male competition (Eikenaar et al. 2009). However, there was a surfeit of quality habitat in our translocated populations, with none of the spatial constraints present on Cousin, as the new islands were unoccupied by Seychelles warblers at the time of translocation. Active female mate choice is only part of sexual selection and mechanisms such as male–male competition can skew social mate choice patterns, whereas sperm competition and cryptic female choice may bias fertilization patterns (Andersson 1994; Jennions and Petrie 2000; Kotiaho and Puurtinen 2007; Løvlie et al. 2013). It is plausible that older or more heterozygous males were more successfully able to compete for females, perhaps through other forms of male–male competition or aggressive coercion (Casalini et al. 2009). Indeed, competitive ability is thought to increase with age in many species (Shutler and Weatherhead 1991; Bose and Sarrazin 2007; Laskemoen et al. 2008). However, our observations showed that males occupied territories and were singing to attract females. Furthermore, females can switch partners readily and initial pairings appear to take time (Komdeur 1996), indicating both a “choosing” period and lack of forced coercion by males. Nevertheless, we cannot rule out that our findings are a result of male–male competition.

Negative results of mate choice studies are generally unlikely to be published (Bernatchez and Landry 2003; Kotiaho and Puurtinen 2007), but such findings are important in establishing the extent to which active mate choice for functional genes such as the MHC occurs. The results of our study suggest that random social pairing with respect to MHC class I characteristics occurs in the Seychelles warbler, regardless of whether or not constraints are present. The occurrence of MHC-dependent EPP (Richardson et al. 2005) suggests an interaction between MHC genes and fertilization patterns that is important in maintaining MHC diversity in this species. However, it may be that the historical constraints on, and costs associated with, social mate choice preferences (Kokko et al. 2003) have prevented the evolution of MHC-dependent social mate choice in the Seychelles warbler, with alternative mechanisms such as age-dependent pairing, promiscuity (driven by whatever mechanism), or postcopulatory selection (e.g., Løvlie et al. 2013) taking precedence. This study highlights that predicting the occurrence of a sexual selection mechanism, that is, MHC-dependent social mate choice, based on seemingly related observations, that is, the MHC-dependent EPP patterns and survival, is not straightforward and that the EPP pattern (Richardson et al. 2005) may reflect mechanisms other than active female choice. There are many potential sexual selection mechanisms that may evolve separately or in concert such as precopulatory or postcopulatory mate choice, coercion, or promiscuity (Andersson 1994; Andersson and Simmons 2006). Understanding how, when, and why particular mechanisms evolve, while others do not, or are observable, while others are not, requires an understanding of the constraints acting on any given species or population. We encourage future studies to focus on how patterns and mechanisms linked to sexual selection may be influenced by the constraints acting within a system.

## SUPPLEMENTARY MATERIAL

Supplementary material can be found at http://www.beheco.oxfordjournals.org/

## FUNDING

The work was supported by a Natural Environment Research Council (NERC) PhD CASE studentship with BirdLife International to D.J.W. supervised by D.S.R. and by NERC grants to D.S.R. (NER/I/S/2002/00712 and NE/F02083X/1). L.B. was supported by an Australian Research Council DECRA fellowship (DE130100174).

## Supplementary Material

Supplementary Data
