# Supplementary material for: Social pairing of Seychelles warblers under reduced constraints: MHC, neutral heterozygosity, and age
Source: Behav Ecol. 2015 Sep 28;27(1):295–303. doi: 10.1093/beheco/arv150 (PMC4718175; doi:10.1093/beheco/arv150)
Supplement: Supplementary Data [file supp_27_1_295__index.html]

Social pairing of Seychelles warblers under reduced constraints: MHC, neutral heterozygosity, and age — Social pairing of Seychelles warblers under reduced constraints: MHC, neutral heterozygosity, and age — Supplementary Data 

# Social pairing of Seychelles warblers under reduced constraints: MHC, neutral heterozygosity, and age

## Supplementary Data

Data files

- Supplementary Data - Supplementary Data
