# Supplementary material for: Social pairing of Seychelles warblers under reduced constraints: MHC, neutral heterozygosity, and age
Source: Behav Ecol. 2015 Sep 28;27(1):295–303. doi: 10.1093/beheco/arv150 (PMC4718175; doi:10.1093/beheco/arv150)
Supplement: Supplementary Data [file supp_arv150_5_Wright_et_al._Supplementary_Material.pdf]

Supplementary Figure (S1): Amino acid sequences translated from MHC Class I exon 3 cDNA sequences of Seychelles warbler (*Ase*) with examples of great reed warbler (*Acar*), scarlet rosefinch (*Caer*), house sparrow (*Pado*), blue tit (*Paca*), green-backed tit (*Pamo*) and chicken (*Gaga*, GenBank accession: X12780). Identity with Seychelles warbler is indicated with a dot, gaps with a tilde, sites corresponding to the peptide binding region with an asterisk, and sites identified as positively selected with shaded boxes.

```
                     10        20        30        40        50        60        70        80
            * * *    |        *|*        |         |       * |**     * |**  *   *|  *   *  |
Ase-UA*1    RLRVSGCELLSDGSVRGSERYGYDGRDFISFELGSGRFVAADSGAEITRRRLEHEGTVAESLTNYLKHECPEWLEKYVGYGQKEL
Ase-UA*2    ..Q.Y.............Y.V..........D.E.................W.....W..R.............Q...E......
Ase-UA*3    V...I.............Y.N..N.......D.E.R....P..........W........GR....T.V.....Q..........
Ase-UA*4    V...Y.............Y.V......................A.......W..D...G.GW.......................
Ase-UA*5    V.............I.....H..............................W..D......F.......................
Ase-UA*6    ..Q.Y.........I.....H................S...A.........W.....W..R.............Q...E......
Ase-UA*7    ....Y.............Y.D..........D...........A.......W.....Q..RW.......................
Ase-UA*8    ....Y.........I...D.D..........D........P..A.......W.....W..R.M......................
Ase-UA*9    ..Q.Y.............Y.V..........D.E.........A.......W....IE..RRM...T.A.....Q..........
Ase-UA*10   .M..I......N..I...Y.N..N.W.................A.......W..D.....RRM....D......QRH.R......
Acar-UA*22  W...H..D.....T...TY.D....Q.L...............A......HW..D.IE..HW....E.K.....QRQIWAEGAGV
Caer*U17    L.W.Y..D......IH..S.L....W.HL..DPK..K..P.N.S..T.GK.W.Q..IEV.RW.....NV...S.R.....RE..R
Pado-UA*19  VQ.LV..D.........FSQD...R......D.E.........A.......W.Q......GW............R...R......
Paca-UA*14  ~~~LY..D............H..............EN.....DA.......W.D.~N...RQE.....V.....R..IR..X~~~
Pamo-509    ~~~.Y..D...N...H.................R.KS.....DA......LW.EK.....GRE.....I.....Q..IS..W.~~
Gaga        VQWMY..DI.EG.PI..YYQMA......TA.DK.TMT.T..VPE.VP.K.KW.~.ESEP.RWK...EET.V...RR..E..KA..
```
